# Proarrhythmic Lipid Inflammatory Mediators: Mechanisms in Obesity Arrhythmias

**DOI:** 10.1002/jcp.70012

**Published:** 2025-02-12

**Authors:** Pegah Bahrami, Kelly A. Aromolaran, Ademuyiwa S. Aromolaran

**Affiliations:** ^1^ Nora Eccles Harrison Cardiovascular Research and Training Institute (CVRTI) University of Utah School of Medicine Salt Lake City Utah USA; ^2^ Division of Cardiothoracic Surgery, Department of Surgery, Nutrition & Integrative Physiology, Biochemistry & Molecular Medicine Program University of Utah School of Medicine Salt Lake City Utah USA

**Keywords:** arrhythmia, eicosanoids, epicardial adipose tissue, GLP‐1 receptor agonists, leukotriene B4, Obesity, SGLT2 inhibitors

## Abstract

The prevalence of obesity and associated metabolic disorders such as diabetes is rapidly increasing; therefore, concerns regarding their cardiovascular consequences, including cardiac arrhythmias, are rising. As obesity progresses, the excessively produced lipids accumulate in unconventional areas such as the epicardial adipose tissue (EAT) around the myocardium. Metabolic alterations in obesity contribute to the transformation of these ectopic fat deposits into arrhythmogenic substrates. However, despite advances in therapeutic approaches, particularly in lowering EAT volume and thickness through sodium‐glucose co‐transporter‐2 (SGLT2) inhibitors and glucagon‐like peptide‐1 (GLP‐1) receptor agonists, obese and diabetic patients still suffer from fatal arrhythmias that may lead to sudden cardiac death. Therefore, an investigation into how unappreciated underlying pathways such as lipid mediators contribute to the transformation of adipose tissues into proinflammatory and arrhythmogenic substrates is of significance. Leukotriene B4 (LTB4) is an eicosanoid derived from arachidonic acid and acts as a lipid mediator. LTB4 has recently been identified to be associated with cardiac ion channel modulations and arrhythmogenic conditions in diabetes. LTB4 increases circulatory free fatty acids (FFAs) and has been associated with adipocyte hypertrophy. LTB4 also interferes with insulin signaling pathways, instigating insulin resistance (IR). In addition, LTB4, as a potent chemoattractant, contributes to the mobilization of circulatory immune cells such as monocytes and promotes inflammatory macrophage polarization and macrophage dysfunction. Thus, this review provides a comprehensive overview of LTB4's underlying pathways in obesity; illustrates how these pathways might lead to alterations in cardiac ion channels, currents, and cardiac arrhythmias; and shows how they might pose a therapeutic target for metabolic‐associated arrhythmias.

**Abbreviations**
5‐LO: 5‐lipoxygenase
AA: arachidonic acid
AKT: protein kinase B
BLT 1/2: leukotriene B4 receptor 1/2
cAMP: cyclic adenosine monophosphate
cPLA2: cytosolic phospholipase A2
EAD: early after‐depolarization
EAT: epicardial adipose tissue
ERK: extracellular signal‐regulated kinase
FOXO1: forkhead box O1
GLP‐1: glucagon‐like peptide 1
GLUT4: glucose transporter type 4
GPCR: G‐protein‐coupled receptor
Gα: Gi alpha subunit
hERG: human ether‐a‐go‐go‐related gene
HFrEF: heart failure with reduced ejection fraction
ICa, L: l‐type calcium current
*I*_*K1*_
: inwardly rectifying potassium current
*I*_*Kr*_
: rapid component of the delayed rectifier potassium current
*I*_*Ks*_
: slow component of the delayed rectifier potassium current
*I*_*Kur*_
: ultra‐rapid outward potassium current
IR: insulin resistance
IRS: insulin receptor substrate
*I*_*to*_
: transient outward potassium current
LTB4: leukotriene B4
MCP‐1: monocyte chemoattractant protein‐1
NF‐κB: nuclear factor kappa B
PI3K: phosphatidylinositol 3‐kinase
PUFA: polyunsaturated fatty acids
SGLT2: sodium–glucose co‐transporter‐2
TdP: Torsade de pointes
TGF: transforming growth factor
TLR: toll‐like receptor
TNF: tumor necrosis factor
VAT: visceral adipose tissue

## 1 Introduction

Obesity, characterized by excessive fat accumulation in the body, poses a global health challenge due to its growing prevalence and strong association with chronic disorders such as insulin resistance (IR), metabolic syndrome, and type 2 diabetes mellitus (T2DM) (Arneth 2024; Ezquerra et al. 2008). The accelerated rise in obesity and obesity‐associated conditions further contributes to elevated risks for the development of cardiovascular adverse events, including arrhythmias and sudden cardiac death (SCD) (Powell‐Wiley et al. 2021). In this regard, the results from the Framingham heart study have shown that the risk of new‐onset atrial fibrillation (AF) is significantly increased in obese individuals (Wang 2004). Furthermore, markers of disturbed ventricular repolarization, including prolonged corrected QT interval (QTc) (Omran et al. 2016) and increased QT dispersion in electrocardiography (ECG) (Dykiert et al. 2024), are observed in obesity. These alterations may lead to ventricular arrhythmia (VA) and SCD (Abdelmegid et al. 2023; K. H. K. Patel et al. 2022; Tikkanen et al. 2022). Despite the significant importance of cardiac arrhythmias and their strong association with SCD in obesity and associated metabolic conditions, proper therapeutic approaches are still an unmet need.

Excessive fat accumulation is the hallmark of obesity, and, interestingly, the localized lipid deposits in obesity are deemed to be the main cause of heightened cardiovascular risk, as higher visceral adipose tissue (VAT) is associated with increased CVD risk, independent from BMI levels (Neeland et al. 2015; Zheng et al. 2023). VAT is considered an ectopic fat tissue, which refers to the accumulation of triglycerides in non‐adipose tissues such as intrabdominal organs (VAT) and the heart (epicardial adipose tissue; EAT), and this is likely the result of adipocyte hypertrophy and subsequent lipolysis in subcutaneous adipose tissues that no longer hold the capacity to store lipids (Hammarstedt et al. 2018). In general, ectopic deposition of lipids interferes with insulin signaling pathways, induces IR, and brings about chronic low‐grade inflammation (L. Liu et al. 2014; Trouwborst et al. 2018). Indeed, EAT samples from patients with coronary artery disease (CAD) undergoing coronary artery bypass grafting have exhibited higher interleukin 1Bꞵ and 6 (IL‐1ꞵ, IL‐6), and tumor necrosis factor‐alpha (TNF‐α) compared with subcutaneous fat depots (Mazurek et al. 2003). IL‐1ꞵ, IL‐6, and TNF‐α have, in turn, exhibited potency in influencing cardiac electrical remodeling, as evidenced by prolonged action potential duration (APD) and QT intervals (markers of delayed repolarization) (Chowdhury et al. 2021; A. Corbin et al. 2014a, 2024b) and increased incidence of early after‐depolarizations (EADs) (Chowdhury et al. 2021), which are indicative of a reduced repolarization reserve and are potential causes of lethal VAs such as torsade de pointes (TdP) (Weiss et al. 2010). The modulatory effects of these cytokines on the APD stem from changes in potassium (K) currents (Aromolaran et al. 2018; A. Corbin et al. 2024a, 2024b), l‐type calcium (Ca) currents (Hagiwara et al. 2007; Krown et al. 1995; Y.‐h. Li and Rozanski 1993), and Ca‐handling proteins (Liao et al. 2021; Monnerat et al. 2016). Among these cytokines, IL‐6 stands out as the most influential in instigating cardiomyocyte electrical vulnerability (Chowdhury et al. 2021), and it exerts a vast range of arrhythmogenic alterations in cardiac ionic channels and currents. In particular, IL‐6 is associated with reduced expression of a surface protein of human ether‐a‐go‐go–related gene (hERG), ERG‐1a, and a decline in the density of its associated current, the rapidly activating component of the delayed rectifier K current (*I*_*K*r)_, in ventricular cardiomyocytes of obese and lipotoxic guinea pigs (A. Corbin et al. 2024a, 2024b). Suppression of *I*_*K*r_ holds significant importance given its capacity to increase the risk of EADs (Liao et al. 2021) and induce TdP (Wit 2018). Hence, investigation of factors that mediate lipotoxicity, ectopic fat formation and its destructive effects, and the underlying mechanisms through which enhanced adipose tissue accumulation may contribute to arrhythmias is essential.

Lipid mediators, particularly leukotrienes, represent an understudied facet within the context of lipotoxicity development and cardiac arrhythmias in obesity. Leukotrienes have been known to be involved in leukocyte infiltration and activation; thus, they are actively involved in both acute and chronic inflammatory diseases (Filgueiras et al. 2015). Leukotrienes comprise cysteinyl leukotrienes and LTB4. The latter can potentially be a target for cardiac arrhythmias, as evidenced by the shortened duration of VAs in rats undergoing ischemia‐reperfusion injury receiving zileuton, an LTB4 and cysteinyl leukotriene inhibitor, rather than montelukast, which is a cysteinyl leukotriene inhibitor (Gonca 2013). LTB4 is strongly associated with lipotoxicity, IR, and a heightened inflammatory profile observed in obesity (P. Li et al. 2015; Spite et al. 2011). Although LTB4's role in adipogenesis and pre‐adipocyte differentiation is controversial (Kae Hirata et al. 2012; K. Hirata et al. 2013), it is well documented that adipose tissue releases significant levels of LTB4 (Spite et al. 2011). Free fatty acids (FFA) are the main form of lipids contributing to cellular dysfunction, and LTB4 is known to increase FFA production in obesity (P. Li et al. 2015). LTB4, through its receptor, interrupts insulin signaling through serine phosphorylation of insulin receptor substrates (IRSs) and attenuates the phosphorylation and subsequent activation of protein kinase B (AKT), which is part of the crucial downstream pathway of insulin signaling (P. Li et al. 2015). Moreover, advanced chemoattraction and migration of monocytes, their subsequent polarization into inflammatory macrophage phenotypes, and secretion of IL‐1ꞵ, IL‐6, and TNF‐α are also extensively observed to occur via LTB4 in obesity and from adipose tissue (Spite et al. 2011; Ying et al. 2017). Collectively, these data suggest a prominent role for LTB4 in arrhythmogenic pathways of obesity. Interestingly, LTB4 levels are elevated in diabetic patients with cardiovascular autonomic neuropathy (CAN) (Santos‐Bezerra et al. 2020), which is a severe and lethal condition comprising autonomic dysfunction leading to cardiac denervation and promotion of long QT intervals, cardiac arrhythmias, and SCD in diabetic patients (Duque et al. 2021). Intriguingly, in vitro administration of LTB4 in guinea pig ventricular myocytes further elucidated a significant decline in *I*_*K*r_ and a reduction in hERG1a protein levels (A. Corbin et al. 2024a, 2024b), demonstrating that LTB4 can directly modulate cardiac electrophysiological changes. Further investigations are required to uncover the precise metabolic mechanisms and cellular signaling pathways through which LTB4 may exert arrhythmogenicity and how they modulate cardiac ionic currents and ion channels in obesity.

**Figure 1:**
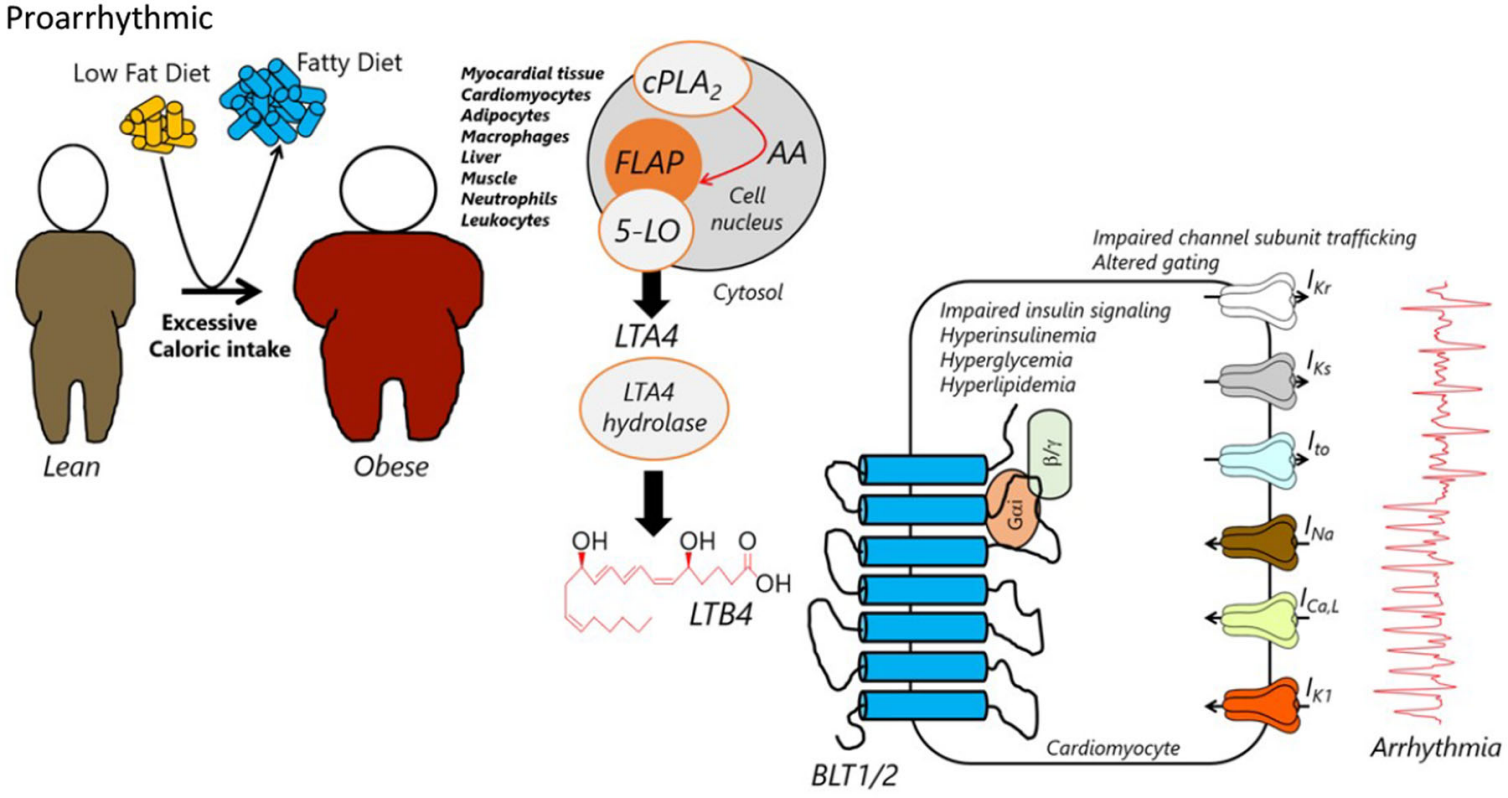
Hypothetical cellular proarrhythmic mechanisms of heightened LTB4 signaling in obesity. High‐fat diet feeding (compared to low fat diet) or excessive caloric intake predisposes to obesity and associated metabolic conditions. We speculate that heightened LTB4 cellular activity in obesity contribute to life threatening arrhythmogenesis, however, precise mechanisms are yet to be identified. LTB4 is generated from arachidonic acid (AA), through cytosolic phospholipase A2 (cPLA2), and the 5‐lipoxygenase (5‐LO) pathway. FLAP (5‐lipoxygenase activating protein) enables the interaction of 5‐LO with the cell membrane and the conversion of AA into leukotriene A4 (LTA4), which is then further converted into LTB4 (the organic structure is shown in *red*) by the enzyme LTA4 hydrolase. The 5‐LO pathway is expressed in myocardial tissue, cardiomyocytes, adipose tissue, macrophages, liver, muscle, neutrophils, and leukocytes. There is evidence that chronically overactive LTB4 directly promotes systemic inflammation and triggers IR, in the liver, muscle, adipose tissue, and cardiomyocytes. LTB4 exerts its effects through the interaction with the G‐protein‐coupled receptor LTB4R1/2 (BLT1/2‐Gαi and β/γ‐linked receptor). BLT1 is the high‐affinity receptor highly expressed in inflammatory and immune cells, whereas BLT2 is a low‐affinity receptor, that is more ubiquitously expressed. While LTB4/LTB4R effects on adipose tissue, liver inflammation, and IR have been broadly studied, comparatively little is known of their effects in heart and/or cardiomyocytes and mechanisms that may underlie increased risk for fatal arrhythmias. Overactive LTB4 signaling may promote arrhythmogenesis and contribute to arrhythmia risk by promoting impaired trafficking and gating mechanisms of major cardiac ion channels including *I*_*Kr*_, *I*_*Ks*_, *I*_*to*_, *I*_*Na*_ (peak and late), *I*_*Ca,L*_ and *I*_*K1*_, further highlighting the importance of multi‐ion channel analyses that may inform the rational development of safer (reduced cardiotoxic effects), antiarrhythmic monotherapy and polytherapy approaches for patients.

Herein, this review aims to provide an overview of potential mechanisms by which LTB4 may influence arrhythmogenicity in obesity and possible therapeutic targets in the underlying pathways involved by exploring LTB4 biosynthesis and how LTB4 is associated with acquired lipotoxicity, IR, and heightened proinflammatory state in obesity (Figure 1). Furthermore, given the significance of EAT in inducing cardiac inflammation, this study also investigates the role of novel antidiabetic medications in reducing EAT size and promoting anti‐arrhythmic alterations in the heart.

## 2 Biosynthesis of Eicosanoids and Relevance to Cardiometabolic Disorders

**Figure 2:**
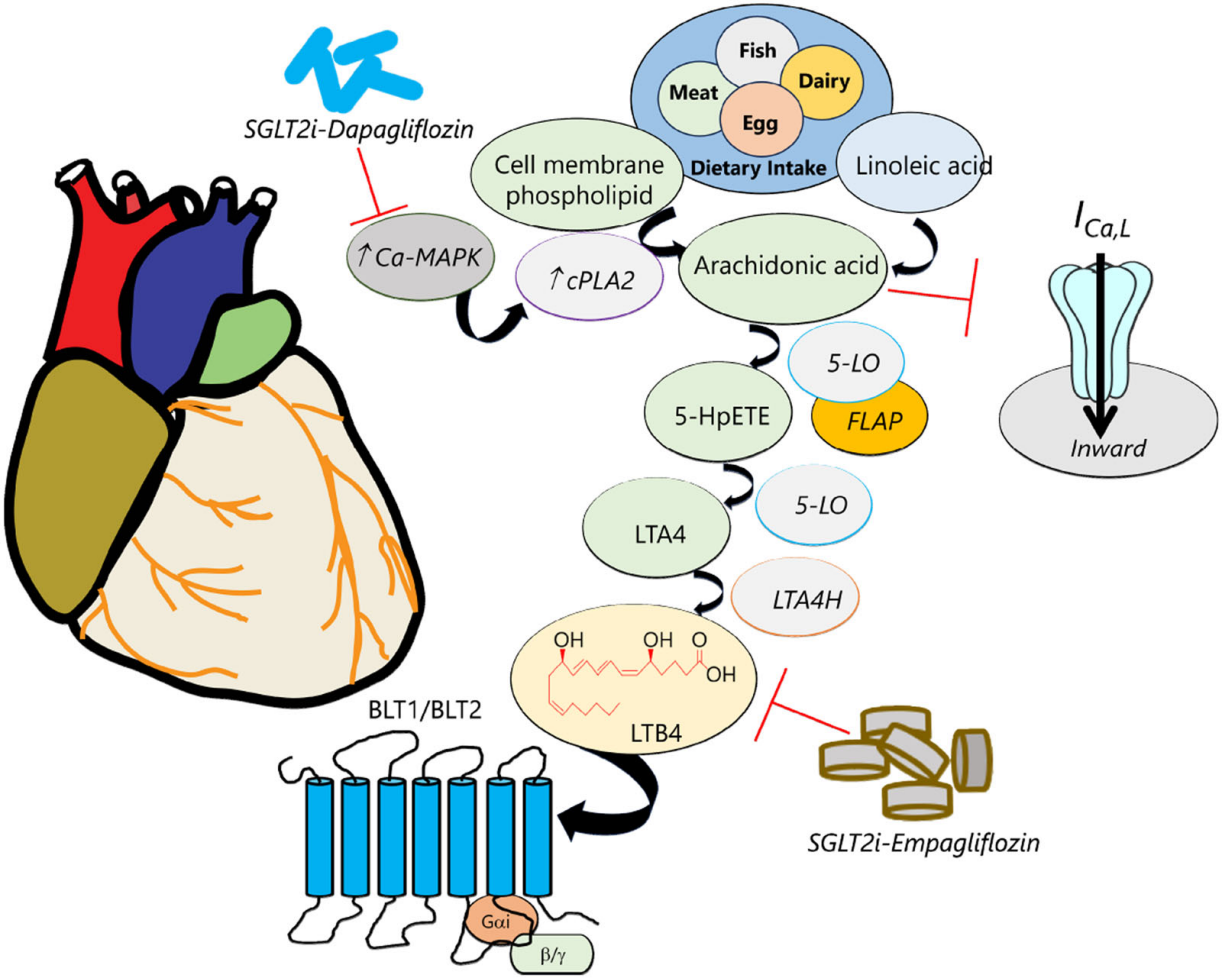
Overview of LTB4 biosynthesis and regulation. High‐fat diet feeding and an increase in adipose tissue promote LTB4 production. LTB4 originates from arachidonic acid (AA) which is an omega‐6 poly‐unsaturated fatty acid. Although AA is not an essential FA and is produced by desaturation and elongation of linoleic acid, it can be considered one if there are insufficient amounts of linoleic acid or the body is not able to convert linoleic acid to AA. AA is also provided through dietary intake of foods rich in omega‐6 like meat and dairy products, fish, and eggs. AA is found in the phospholipid membrane of various cells throughout the body and is released from the phospholipid membranes by cytosolic phospholipase A2 (cPLA2), a calcium (Ca) sensitive enzyme that also serves as a substrate for the mitogen‐activated protein kinase (MAPK). Interestingly, phosphorylation of cPLA2 by MAPK can be inhibited by dapagliflozin, in turn curbing the release of AA and reducing associated downstream pathways. Even though AA plays a significant role in maintaining cellular function and energy, excessive amounts of AA may promote a reduced Ca influx through l‐type Ca channels leading to enhanced adipogenesis manifested as an increase in the size and number of adipocytes. Once released, AA is converted to leukotriene A4 (LTA4) through a two‐step process catalyzed by 5‐lipoxygenase (5‐LO). Subsequently, LTA4 is converted into LTB4 by LTA4 hydrolase (LTA4H), and LTB4 will exert its downstream effects through its receptors (BLT1/2). There is increasing evidence that suggests that SGLT2 inhibitors (SGLT2i, dapagliflozin and empaglifozin) may target Ca‐MAPK and directly LTB4 levels to normalize LTB4 levels in the setting of obesity/diabetes. AA, arachidonic acid; cPLA2, cytosolic phospholipase A2; 5‐HpETE, 5 (S) hydroperoxy‐6‐trans‐8,11,14‐cis‐eicosatetraenoic acid; *I*_*Ca,L*_, l‐type Ca current; 5‐LO, 5‐lipoxygenase; LTA4, leukotriene A4; LTB4; leukotriene B4; MAPK, mitogen‐activated protein kinase.

Eicosanoids encompass a large family of active lipid derivatives acting as signaling molecules, and they originate from polyunsaturated fatty acids (PUFAs) (Araújo et al. 2018). Arachidonic acid (AA) is an omega‐6 (ω‐6) PUFA and the main substrate of leukotrienes and prostanoids, each of which are derived from AA through lipoxygenase and cyclooxygenase enzymes, respectively (Wan et al. 2017). AA, which is provided either through dietary intake or the desaturation and elongation of an essential FA called linoleic acid, is found in the phospholipids of cellular membranes in various organs throughout the body, with the liver being a main site of production (Bermúdez et al. 2021; Tallima and El Ridi 2018) (Figure 2). In the heart, AA is likely deposited in both phospholipid and triacylglycerol pools (Linssen et al. 1992) and it has been reported that exposure to extracellular AA can directly inhibit *I*_Ca,L_ channel current by interfering with its gating mechanisms in ventricular myocytes (S. J. Liu 2007). AA is released from phospholipids by phospholipase A2 (PLA2), particularly by the cytosolic PLA2 (cPLA2) family (Bermúdez et al. 2021; Khan and Ilies 2023), and it is proposed that AA itself subsequently leads to the redistribution and translocation of cPLA2 towards the endoplasmic reticulum, creating a positive feedback loop between the enzyme and AA (Wooten et al. 2008). cPLA2 is a Ca‐dependent enzyme that is ubiquitously expressed in various tissues, including cardiomyocytes (Ait‐Mamar et al. 2005; Leslie 1997; Mohamed et al. 2014), where it is translocated from the cytosol to the sarcoplasmic reticulum (SR) upon activation by heightened intracellular Ca concentrations and stimulation of mitogen‐activated protein kinases (MAPKs) (Magne et al. 2001). MAPK‐induced cPLA2/AA signaling has been associated with increased Ca restoration in the SR (Magne et al. 2001), and although this signaling is restricted upstream by cyclic adenosine monophosphate (cAMP), once AA is produced, the Ca accumulation effect of cPLA2/AA works synergistically with the cAMP‐induced Ca release from the SR, allowing for ventricular cardiomyocyte contraction (Pavoine and Defer 2005).

The cPLA2/AA pathway is involved in adipocyte maturation and expansion. In vitro analysis of preadipocytes has revealed that cPLA2α and AA instigate early maturation of these cells into adipocytes (Peña et al. 2016). Furthermore, high‐fat diet (HFD)‐fed mice lacking cPLA2α have exhibited reduced white fat tissue mass with smaller adipocyte cells, indicating that the cPLA2α/AA pathway contributes to obesity‐associated adipose tissue expansion by increasing both the number and size of adipocytes (Peña et al. 2016). The adipocyte‐derived adipokine, leptin, has been shown to augment cPLA2α expression at the gene and protein levels through its receptor, OB‐R, in lung cells (Hsu et al. 2015), potentially forming a vicious cycle between adipose tissue and the cPLA2α/AA pathway. However, there is a lack of evidence on whether the same pattern exists in cardiomyocytes. It can be suggested that future studies evaluate whether ventricular cardiomyocytes from ob/ob or db/db mice, which are known mutant animal models of severe obesity and diabetes and exhibit deficient or resistant leptin phenotypes, respectively (Suriano et al. 2021), express elevated levels of cPLA2α and AA and how these changes modulate cardiac ionic channels and Ca handling proteins. This notion is emphasized by recent evidence demonstrating that Dapagliflozin, an anti‐T2DM and sodium–glucose co‐transporter‐2 (SGLT2) inhibitor medication, is reported to decrease ultra‐rapid outward potassium current (*I*_*Kur*)_, *I*_Kr_, and late sodium current (*I*_*Na)*_ and increase inwardly rectifying potassium current (*I*_*K1*)_ (Müller et al. 2024; Philippaert et al. 2021), in addition to alleviating diabetic retinopathy by abrogating the cPLA2 phosphorylation by MAPK, thereby reducing AA (Hu et al. 2022). Further, analysis by Pena et al. has shown that co‐administration of indomethacin, a cyclooxygenase inhibitor, to preadipocytes treated with AA could only partially suppress the adipogenic transformations induced by AA (Peña et al. 2016), indicating the involvement of other downstream pathways.

Once released, AA is processed via three distinct pathways leading to prostanoids, hydroxyeicosatetraenoic (HETE) and epoxyeicosatrienoic (EET) acids, and leukotrienes (Wan et al. 2017). To synthesize LTB4, AA is metabolized to 5(S)hydroperoxy‐6‐trans‐8,11,14‐cis‐eicosatetraenoic acid (5‐HpETE) and then LTA4 in a two‐step manner by 5‐lipoxygenase (5‐LO or ALOX5) and its activator protein, 5‐lipoxygenase‐activating protein (FLAP) (Wan et al. 2017). LTA4 is finally converted into LTB4 by LTA4 hydrolase (LTA4H) (Wan et al. 2017). Parallel to LTB4 production, AA is also metabolized into cysteinyl LTs and lipoxins (B. Wang et al. 2021). Alterations in any stage of this pathway can potentially alter LTB4 levels. 5‐LO is a crucial enzyme and mediator in LTB4 production, and its deficiency or inhibition leads to a decline in LTB4 synthesis, alleviating the overall inflammatory response (Filgueiras et al. 2015). It is known that dysregulations in insulin signaling can autonomously promote the 5‐LO/LTB4 pathway at the 5‐LO level by bypassing the inhibitory effect of insulin on Forkhead box O1 (FOXO1), a downstream transcription factor that is translocated from the nucleus to the cytosol upon phosphorylation in the insulin pathway, without a concomitant increase in inflammatory cytokines and immune cells (Hosooka et al. 2020; Teaney and Cyr 2023). Intriguingly, elevated levels of nuclear FOXO1 have been observed in the myocardium of T1DM rats (Yan et al. 2020). Furthermore, muscle homogenates of T1DM mice lacking 5‐LO exhibited elevated insulin receptor gene levels despite low insulin concentrations (Guimarães et al. 2019). Even though these studies are focused on T1DM, IR is also commonly observed in obesity and T2DM. These findings highlight the role of 5‐LO in the intricate relationship between LTB4 and insulin; however, whether there is indeed a vicious cycle that starts with dysregulations in insulin signaling, leads to 5‐LO activation by FOXO1, and ends with reduced insulin receptors due to LTB4 needs to be determined.

Lastly, as LTB4 is released, its pathway is initiated by LTB4 binding to its G protein‐coupled receptors, BLT1 and BLT2 (Tager and Luster 2003). BLT2 is ubiquitously expressed in the body, holds a lower affinity for LTB4, and is generally more correlated with non‐cardiovascular inflammation than BLT1 (Tager and Luster 2003; Yokomizo and Shimizu 2023). On the other hand, the LTB4/BLT1 axis is directly involved in cardiac lipotoxicity, IR, and inflammation (P. Li et al. 2015).

## 3 The Role of LTB4 in Lipotoxicity and Alterations in Adipose Tissue

Lipotoxicity has a role in obesity‐induced electrical remodeling and cardiac arrhythmias. Patch clamp analysis of palmitic acid (PA)‐induced lipotoxic guinea pig ventricular cardiomyocytes revealed an increase in APD and QTc and a higher susceptibility to VAs (Chowdhury et al. 2021). Similarly, HFD‐ and PA‐fed preclinical models exhibit higher arrhythmogenicity, as evidenced by their higher numbers of spontaneous beats and elevated AF inducibility upon stimulation (Chan et al. 2019; Martinez‐Mateu et al. 2019). Given the prominent rise in LTB4 levels in the liver, muscle, and adipose tissue of HFD‐fed mice in a study by P. Li et al. (2015) and in the plasma samples of ob/ob T2DM mice in a recent study by Corbin et al. (2024a, 2024b), it can be proposed that LTB4, at least partly, may be responsible for these electrical alterations in HFD‐fed and lipotoxic hearts. This is further confirmed by the marked increase in LTB4 in PA + glucose‐treated guinea pig ventricular myocytes, as also found in the study by Corbin et al. (2024a, 2024b). To uncover this hypothesis, understanding the relationship between LTB4 and obesity‐related changes in adipose tissue is crucial.

Given the proximity of EAT to the myocardium, the secretion of proinflammatory cytokines by EAT (Kawai and Akira 2007), the modulation of cardiac ionic currents by cytokines (Kiran Haresh Kumar Patel, Hwang, Se Liebers, & Ng, 2021), and increases in FFAs, it would be beneficial to evaluate the role of LTB4 in EAT formation and its arrhythmogenicity given that LTB4 is associated with higher serum FFAs (P. Li et al. 2015) and contributes to the release of proinflammatory cytokines and FFAs (Ying et al. 2017). Although the secretion of LTB4 from EAT is yet to be elucidated, since enzyme‐linked immunosorbent assay (ELISA) assessments have demonstrated elevated LTB4 levels in the VAT of obese mice (Ying et al. 2017), it can be hypothesized that EAT also secretes a substantial amount of LTB4. EAT, which is naturally present around the myocardium, is an interesting type of white adipose tissue that also holds brown fat characteristics by promoting thermogenesis (Gianluca Iacobellis 2022). Under pathologic conditions such as obesity and T2DM, EAT loses its brown‐like features and transforms from its cardioprotective nature to an inflammatory substrate (M. Packer 2018). One of the changes evident in EAT as it becomes a site of excessive fat accumulation under the influence of metabolic inflammation and IR is the presence of hypertrophic adipocytes (Kologrivova et al. 2023; Naryzhnaya et al. 2021), which may resonate with the findings of the study by Mothe‐Satney et al (2012). on the positive correlation between the increased production of LTB4 from adipocytes and adipocytes’ size. Thus, LTB4 may play a role in the adipocyte hypertrophy observed in EAT. Targeting LTB4 could, thereby, mitigate the arrhythmogenic effects of EAT by reducing its overall volume.

## 4 The Role of LTB4 in IR

IR has been identified as a cornerstone for diabetes‐induced cardiac diseases (Fan et al. 2024) and is known to be the bridge between obesity and lipotoxicity (Reaven 2011). Cell‐based analyses have demonstrated that insulin is involved in the expression and synthesis of pore‐forming and regulatory protein subunits of *I*_*Ks*_ and transient outward potassium current (*I*_*to*_) (Lengyel et al. 2007; Torres‐Jacome et al. 2013); therefore, it is not surprising that disturbances in insulin signaling may lead to cardiac arrhythmias. Moreover, IR has been associated with ventricular (Shah et al. 2013; Yang et al. 2019; Yethindra et al. 2021) and atrial remodeling (Chan et al. 2019). Thus, it is crucial to understand the direct and indirect roles of LTB4 in IR stimulation.

Given the proinflammatory nature of LTB4 and the significant role of augmented infiltration of cytokines such as TNF‐α, IL‐1ꞵ, and IL‐6 in IR (Bradley et al. 2022; Gao et al. 2014; Kurauti et al. 2017; Olson et al. 2012; Plubell et al. 2018; Uysal et al. 1997), it could be proposed that LTB4 indirectly promotes IR cytokines through inflammatory cascades. However, LTB4 can also directly reduce cellular insulin sensitivity (Hosooka et al. 2020; P. Li et al. 2015), and it may even play a more significant role in IR than inflammatory pathways, since in vivo depletion of macrophages with clodronate in obese mice treated with a BLT1 inhibitor resulted in a greater improvement in glucose tolerance compared with those receiving only clodronate (P. Li et al. 2015).

Alterations in insulin signaling and function are brought about by dysregulations of the insulin receptor, IRS, and the insulin signaling pathways, including changes in the phosphatidylinositol 3‐kinase (PI3K)/protein kinase B (Akt) and MAPK/extracellular signal‐regulated kinases 1/2 (ERK1/2) pathways (Fan et al. 2024). Phosphorylation of Akt by PI3K activates it, leading to various cellular functions that include glucose uptake through the translocation of glucose transporter 4 (GLUT4) (Gabbouj et al. 2019). Additionally, PI3K pathways are associated with protein trafficking and gating of the cardiac Na, l‐type Ca, and ERG channels (Ballou et al. 2015). Western blot analyses revealed a decline in insulin‐induced phosphorylated Akt protein levels in LTB4 + insulin‐treated myocytes and hepatocytes compared with cells treated solely with insulin (P. Li et al. 2015). The same study demonstrated that this finding correlated with reduced glucose transportation and GLUT4 expression by insulin (P. Li et al. 2015), indicating that LTB4 suppresses insulin‐mediated Akt phosphorylation and the subsequent GLUT4 translocation. Further, it was revealed that LTB4 exerts these effects through BLT1 (P. Li et al. 2015). Since reduced translocation of GLUT4 in the atria of HFD‐fed IR mice has been shown to be parallel with high susceptibility to AF (Maria et al. 2018), it can be postulated that LTB4 may be involved in obesity‐induced AF through this mechanism (Ballou et al. 2015). However, PI3K inhibition has not resulted in suppression of insulin's ability to regulate *I*_*to*_ currents; rather, the ERK1/2 inhibitor PD98059 can diminish the insulin‐mediated increase in *I*_*to*_ density (Shimoni et al. 1998). Modulation of the MAPK/ERK pathway by LTB4 has not yet been well demonstrated, but the pathway's role in *I*_*to*_ regulation prompts the need to evaluate the influence of LTB4 in modulating this pathway.

LTB4 has been found to block IRS function through JNK‐induced serine phosphorylation of IRS1 and IRS2 in myocytes and hepatocytes of obese HFD‐fed mice (P. Li et al. 2015). Notably, IRS1 and IRS2 play crucial roles in maintaining cardiac energy (Guo and Guo 2017). P. Li et al. (2015) demonstrated that the LTB4‐mediated serine phosphorylation of IRS1 was mediated through the binding of LTB4's G‐protein‐coupled receptor, BLT1, to Gαi proteins, a family of heterotrimeric G protein alpha subunits. Notably, GPCR signaling through Gαi suppresses cAMP production (Y. Li and Anand‐Srivastava 2023), thereby potentially dampening its effect on promoting and inhibiting the expression of M2 and inflammatory macrophages (M1), respectively (Negreiros‐Lima et al. 2020; Polumuri et al. 2021). Furthermore, a switch in the Gα family subunit towards Gαi led to the activation of nuclear factor kappa B (NF‐κB), a main regulator of innate immunity and inflammation (Liu et al. 2017), and it augmented proinflammatory cytokine secretion, confirming the proinflammatory behavior of the Gαi pathway (Zhang et al. 2018). Gαi has exhibited isomer‐specific effects on cardiac electrical remodeling (Nagata et al. 2000; Ruan et al. 2007). Inhibition of Gαi1 led to a decline in the frequency of VAs and phospholamban (PLB) phosphorylation, along with ameliorated AP prolongation (Ruan et al. 2007), whereas deletion of Gαi2 failed to benefit from an anti‐adrenergic effect to reduce the isoproterenol‐induced augmented Ca transient in ventricular cardiomyocytes of adult mice (Nagata et al. 2000). The specific details of how LTB4 alters Gαi isomers in cardiac tissue and how the LTB4/BLT1/Gαi/IRS pathway may modulate cardiac ions and channels are yet to be determined. Investigating these gaps might assist in utilizing anti‐LTB4 medications as anti‐arrhythmic agents in IR.

## 5 The Role of LTB4 in Metabolic Inflammation

In general, inflammation is capable of inducing cardiac arrhythmias either directly by promoting fibrosis and electrical changes in cardiac fibroblasts and cardiomyocytes or indirectly through systemic effects by promoting lipotoxicity, IR, and autonomous nervous system activity (Lazzerini et al. 2023). Heightened levels of LTB4 in obesity and T2DM have been found to instigate systemic inflammation and macrophage reprogramming toward a proinflammatory phenotype (Bonyek‐Silva et al. 2020; Spite et al. 2011). It has been widely suggested in preclinical obesity models that the 5‐LO/LTB4/BLT1 pathway positively influences the secretion of IL‐6, TNF‐α, and IL‐1β (Bonyek‐Silva et al. 2020; Horrillo et al. 2010; P. Li et al. 2015; Mothe‐Satney et al. 2012; Spite et al. 2011; Ying et al. 2017). Understanding the putative mechanisms governing LTB4's contribution to heightened cytokine secretion is crucial, as it provides insight into potential therapeutic approaches.

Monocytes are members of the innate immune system that are found to migrate to adipocytes from the bloodstream in obesity, where they are subsequently polarized into M1 macrophages, which initiates a cascade leading to proinflammatory cytokine secretion and, eventually, IR (Y. Liu et al. 2020). Obesity, HFD, and diabetes have been associated with increased concentrations of peripheral monocytes (Pant et al. 2024; Spite et al. 2011; Takahashi et al. 2003). LTB4 is a potent chemotaxis agent and a critical element in monocyte trafficking (Huang et al. 2004). Flow cytometry analyses have shown an augmented expression of BLT1 on peripheral proinflammatory CD11b+ monocytes in obese mice (Spite et al. 2011). Interestingly, Spite et al. (2011) revealed that BLT1 deletion (BLT1^−/−^ mice) results in the total elimination of monocyte expansion in obesity, indicating that the LTB4/BLT1 pathway is the key regulatory mechanism behind the enhanced expression of monocytes in metabolic disorders. Furthermore, an increase in the expression of monocyte chemoattractant protein‐1 (MCP‐1) at both the transcriptional and post‐transcriptional levels has been associated with LTB4 in the same BLT1‐dependent manner (Choi et al. 2021; Huang et al. 2004; P. Li et al. 2015; Spite et al. 2011). MCP‐1 is a small cytokine and an effective chemoattractant for circulating monocytes; its plasma levels are increased in T2DM patients (Piemonti et al. 2009), and its protein expression is elevated in lipotoxic cardiomyocytes, as evidenced by its high protein expression in PA‐induced lipotoxic HL‐1 cardiomyocytes (Ren et al. 2024). Intriguingly, MCP‐1, in turn, induces LTB4 formation, creating a positive feedback loop and exacerbating the inflammatory state of obesity (Silva et al. 2002). This intricate relationship among LTB4, BLT1, and MCP‐1 could be an intriguing therapeutic target for cardiometabolic‐associated arrhythmia given the heightened levels of MCP‐1 protein in the atria of T2DM mice, as well as their attribution to ryanodine receptor 2 (RyR2)—a Ca‐handling protein—activation and AF susceptibility (Ren et al. 2024; Zhou et al. 2024). Nevertheless, LTB4's effects are not limited to monocytes’ mobilization, as an attenuated level of M1 macrophages has been observed in BLT1‐deficient mice (Spite et al. 2011). Indeed, LTB4 induction in RAW264.7 macrophage cells also led to overexpression of the CD86 and CD11c genes (Gong et al. 2021), which are markers of M1 macrophages (Smith et al. 2016), thus confirming the role of LTB4 in the phenotypic switch of macrophages. The CD11c marker, in particular, is found to be overly expressed in the adipose‐tissue‐derived macrophages of obese preclinical models, whereas their lean counterparts have expressed higher M2 specific markers comprising chitinase‐like protein Ym1 and arginase 1 (Arg1) (Lumeng et al. 2007). This trend of overexpressed Ym1 and Arg1 is consistent with the findings in 5‐LO knockout T1DM mice, which, compared with T1DM mice that were able to produce LTB4, did not exhibit an elevation in IL‐6 levels (Guimarães et al. 2019). Moreover, LTB4 also exerts destructive alterations in macrophages, leading to enhanced glycolysis and reduced mitochondrial function (Ramalho et al. 2019). Increased lipid droplet content was observed in macrophages in T1DM mice, and it was abolished in mice treated with a BLT1 inhibitor (Ramalho et al. 2019). Likewise, LTB4 treatment in guinea pigs’ ventricular cardiomyocytes increased the number and size of lipid droplets in these cells (A. Corbin et al. 2024a, 2024b).

LTB4 further influences the release of inflammatory cytokines by modulating their underlying cellular mechanism. An innate immunity‐associated pathway involving toll‐like receptors (TLRs) governs a major part of IL‐6 production. TLRs are transmembrane pattern recognition receptors that, by binding to pathogen‐associated molecular patterns (PAMPs), eventually activate the myeloid differentiation primary response 88 (MYD88) protein complex, which then triggers NF‐κB (Kawai and Akira 2007), a pathway that is known to be augmented in obesity and lipotoxicity (Qian et al. 2024). The 5‐LO/LTB4 pathway plays a significant role in modulating the TLR pathway, and both 5‐LO and FLAP inhibition resulted in abrogated TLR‐2‐, ‐3‐, and ‐4‐mediated phagocytosis (Ebert et al. 2020); of these, TLR‐2 and ‐4 were found to be elevated in EAT tissue from CAD patients (Vianello et al. 2016). While no direct relationship has been found between LTB4 and TLR expression (Gaudreault et al. 2012), a downstream protein of this pathway known as TGF‐β‐activated kinase 1 (TAK1) is phosphorylated by LTB4, leading to NF‐κB overexpression and cytokine release (Gaudreault et al. 2012). In addition, LTB4 increases the expression of MyD88 through positive regulation of miR‐155 (Z. Wang et al. 2014), thus further contributing to this pathway. This pathway is particularly important given the higher levels of IL‐6 in the EAT of diabetic patients with AF (Takano et al. 2023). The same cohort of patients demonstrated that IL‐6 is decreased in patients taking SGLT2 inhibitors (Takano et al. 2023), making this pathway a practical therapeutic target.

## 6 Targeting Eat as a Novel Therapeutic Approach

**Figure 3:**
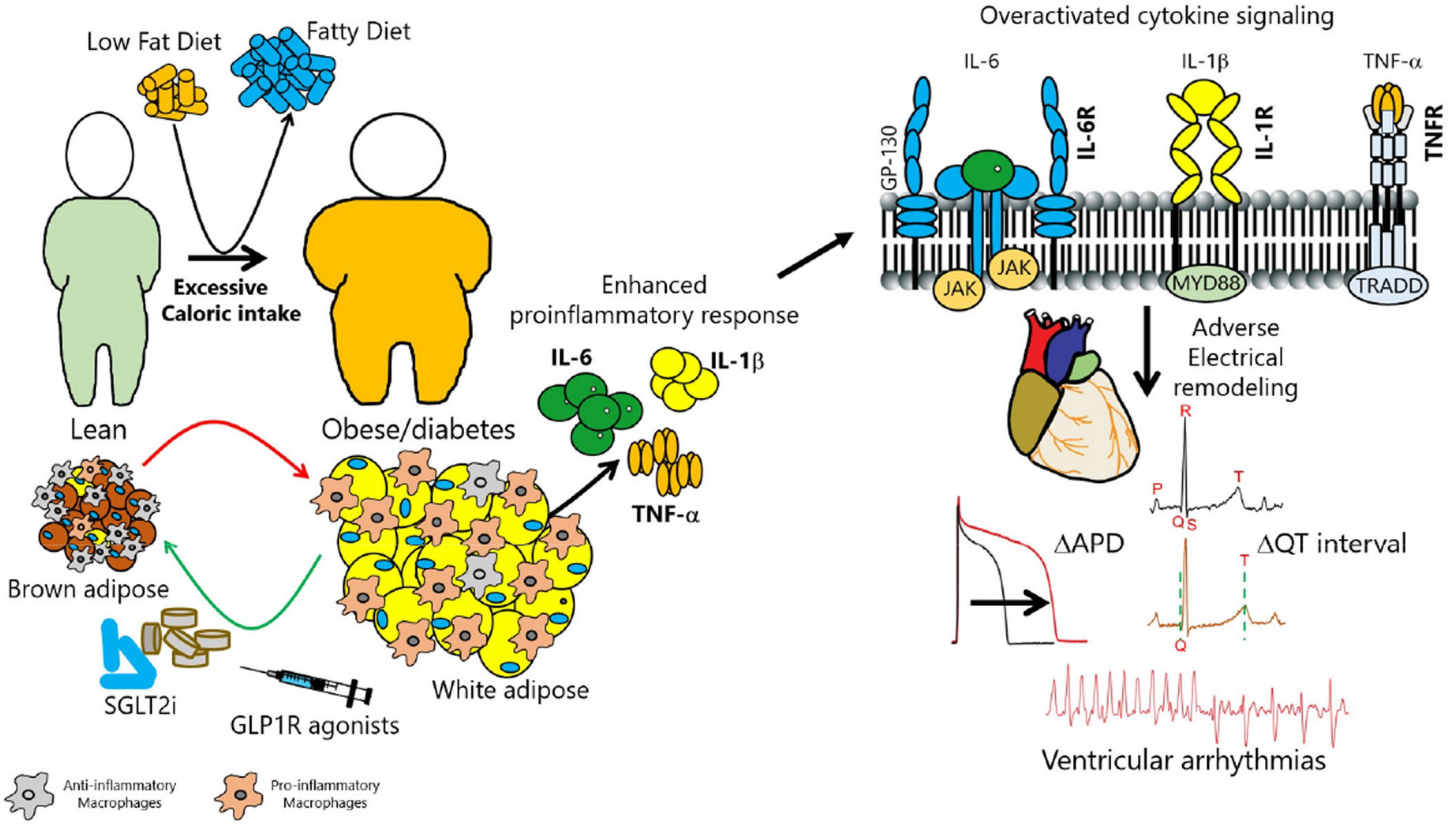
Arrhythmogenic changes in EAT and therapeutic interventions. Epicardial adipose tissue (EAT) is mostly composed of white adipose tissue, however, under physiological conditions, EAT maintains characteristics of brown adipose tissue by regulating thermogenesis and thus providing cardioprotection through creating thermal and energy balance. In contrast, pathologic conditions like obesity and diabetes disrupt this balance which leads to the formation of potentially arrhythmogenic white adipose features, and heightened infiltration/accumulation of proinflammatory macrophages. Subsequently, EAT becomes an inflammatory substrate secreting an excessive amount of proinflammatory cytokines including IL‐6, IL‐1ꞵ, and TNF‐α, and overactivating cytokine signaling thus increasing the susceptibility to cardiac arrhythmias manifested as cellular proarrhythmic mechanisms including pathological action potential duration prolongation, leading to QT prolongation that increases sudden cardiac death risks. Advances have been made to reduce the pathologic effects of EAT. SGLT2 inhibitors (empagliflozin, dapagliflozin, canagliflozin, luseogliflozin) have shown promising outcomes in reducing EAT volume. Similarly, GLP1‐RAs (semaglutide, dulaglutide, liraglutide, and exenatide) have successfully decreased EAT thickness seemingly by binding to GLP1 receptors on the EAT. Furthermore, since a correlation has been found between GLP1 expression and genes involved in the browning of adipose tissue, it could be concluded that GLP1‐RAs might enhance brown adipose tissue characteristics and thereby induce cardioprotective effects. ΔAPD, change in action potential duration; EAT, epicardial adipose tissue; GLP‐1 RA, glucagon‐like peptide‐1 receptor agonist; IL‐6, interleukin 6; IL‐1ꞵ, interleukin 1ꞵ; ΔQ interval, change in QT interval (denoted by the green dotted lines); SGLT2i, sodium‐glucose co‐transporter‐2 inhibitor; TNF‐α, tumor necrosis factor α.

EAT has emerged as a major contributing factor for AF, which is partly due to the secretion of inflammatory cytokines and FFAs (Gianluca Iacobellis 2022). Interestingly, conventional calorie‐decreasing diets have not yielded positive results in reducing EAT (Milton Packer 2019); however, novel antidiabetic agents consisting of SGLT2 inhibitors (empagliflozin, dapagliflozin, canagliflozin, and luseogliflozin), and glucagon‐like peptide‐1 (GLP‐1) receptor agonists (liraglutide, semaglutide, exenatide, dulaglutide) have been reported to lower EAT (Supporting Information S1: Table S1)(Myasoedova et al. 2023; Requena‐Ibáñez et al. 2021) (Figure 3). These medications have also, to some extent, exhibited modulatory effects on cardiac ion channels, currents, and Ca‐handling proteins (Supporting Information S1: Table S2) (Müller et al. 2024; Philippaert et al. 2021). Therefore, they may be considered promising antiarrhythmic agents in these metabolic conditions.

### 6.1 SGLT2 Inhibitors

SGLT2 inhibitors are a family of anti‐T2DM medications that function by inhibiting glucose reabsorption in renal tubules and have shown promising results in reducing CVD‐related morbidity and mortality (Buse et al. 2020). Among SGLT2 inhibitors, empagliflozin has demonstrated the lowest risk of cardiovascular adverse events (Jiang et al. 2022) and has strong associations with cardiac electrical remodeling, as evidenced by its ability to increase *I*_*Kr*_, *I*_*Ks*_, and *I*_*CaL*_ (Karpushev et al. 2022; Lee et al. 2019), decrease late *I*_*Na*_ (Hegyi et al. 2022; Lee et al. 2019; Philippaert et al. 2021; Wen et al. 2024) densities, and modulate Ca‐handling proteins (Lee et al. 2019; Moellmann et al. 2020; Wen et al. 2024), which ultimately leads to reduced APD (Karpushev et al. 2022; Lee et al. 2019; Mira Hernandez et al. 2024) and attenuated cardiac arrhythmias (Mira Hernandez et al. 2024). Although the administration of this drug to T1DM rats demonstrated a reduction in lipid depositions in cardiac tissue (Xi et al. 2022), contrasting data exist regarding empagliflozin's role in reducing EAT volume (Gaborit et al. 2021; Requena‐Ibáñez et al. 2021). While empagliflozin effectively reduced EAT volume in non‐diabetic patients with heart failure with a reduced ejection fraction (HFrEF) (Requena‐Ibáñez et al. 2021), it did not yield significant results in T2DM patients receiving 10 mg/day of empagliflozin for 12 weeks (Gaborit et al. 2021). Even though both studies used the same dosage of empagliflozin and the same imaging technique (MRI), in the first study, an MRI was conducted 6 months from the baseline, as opposed to the 3‐month study timeline for diabetic patients. Similarly, proton magnetic resonance spectroscopy (^1^HMRS) imaging in mice receiving a high‐fat and high‐sucrose diet coupled with oral administration of empagliflozin demonstrated no changes in the myocardial triglyceride content compared with their counterparts not receiving the drug (Gaborit et al. 2021). Interestingly, in contrast to EAT, hepatic fat depots were effectively reduced by empagliflozin in the same study (Gaborit et al. 2021). Further studies are needed to elucidate the long‐term effects of empagliflozin in diabetic patients.

On the other hand, short‐term (1 month) and long‐term (6 months) administration of dapagliflozin, another SGLT2 inhibitor with known anti‐arrhythmic impacts on cardiac ionic currents (Müller et al. 2024; Paasche et al. 2024; Philippaert et al. 2021), exhibited mostly promising outcomes in lowering EAT in T2DM patients with or without CAD (Braha et al. 2019; Cinti et al. 2023; Iacobellis and Gra‐Menendez 2020; Macías‐Cervantes et al. 2024; Sato et al. 2020; Song et al. 2023). Similarly, canagliflozin and luseogliflozin also demonstrated promising outcomes regarding lowering the EAT volume after 6 and 3 months of administration, respectively (Bouchi et al. 2017; Yagi et al. 2017).

The underlying mechanisms through which SGLT2 inhibitors act against EAT differ among these medications. While dapagliflozin induces adipocyte maturation and increases glucose uptake through the upregulation of GLUT4 protein in mature adipocytes (Díaz‐Rodríguez et al. 2018), empagliflozin reduces the size of lipid droplets by targeting the SGLT2 receptor expressed in pre‐adipocytes (Takano et al. 2023). Surprisingly, in vitro administration of empagliflozin in cardiomyocytes pretreated with doxorubicin or ipilimumab and high glucose attenuated the LTB4 levels (Maurea et al. 2019; V. Quagliariello et al. 2020; Vincenzo Quagliariello et al. 2021), which was consistent with the lower levels of IL‐1β and IL‐6 in empagliflozin + doxorubicin‐treated cardiomyocytes (Vincenzo Quagliariello et al. 2021). Therefore, it would also be beneficial to evaluate the role of LTB4 modulation by these drugs in lowering EAT volume and thickness.

### 6.2 GLP‐1 Receptor Agonists

GLP‐1 receptors, which are similar to LTB4 receptors, are members of the GPCR family and are widely expressed in various tissues in the body, including the pancreas, heart, adipose tissue, and EAT in particular (Baggio et al. 2018; Cantini et al. 2016; G. Iacobellis et al. 2017). GLP‐1 receptor agonists mimic GLP‐1 and bind to and activate the GLP‐1 receptor, which, in turn, instigates and suppresses insulin and glucagon secretion, respectively (Cantini et al. 2016). The benefits of GLP‐1 receptor agonists extend beyond their ability to enhance glycemic control, as liraglutide, semaglutide, and dulaglutide exhibited a decline in the risk of various components of major adverse cardiovascular events (MACE) (Gerstein et al. 2019; Marso et al. 2016; Marso Steven et al. 2016). In addition, a recent meta‐analysis indicated that GLP‐1 receptor agonists hold a stronger ability to reduce EAT size than SGLT2 inhibitors do (Myasoedova et al. 2023). There is evidence of a dose‐dependent reduction in EAT thickness following treatment with semaglutide and dulaglutide for 3 months (G. Iacobellis and Villasante Fricke 2020). While the changes in EAT demonstrated by these two drugs were not correlated with hemoglobin A1c levels (HbA1c)—a glycosylated hemoglobin made by binding hemoglobin with glucose (G. Iacobellis and Villasante Fricke 2020)—a strong correlation was found between these two parameters in the 6‐month administration of liraglutide (G. Iacobellis et al. 2017). A significant reduction in EAT thickness was reported in T2DM patients receiving liraglutide + metformin compared with those receiving metformin alone (G. Iacobellis et al. 2017). Moreover, liraglutide and exenatide have shown promising outcomes in terms of reducing EAT thickness in T2DM patients with uncontrolled diabetes (Dutour et al. 2016; Morano et al. 2015; Zhao et al. 2021). Nevertheless, there are contrasting data from studies indicating no significant changes in EAT as a result of liraglutide (Bizino et al. 2020; van Eyk et al. 2019), but these studies consisted of small patient populations.

The cellular mechanisms shaping the effects of GLP‐1 receptor agonists on EAT are yet to be demonstrated. It can be postulated that GLP‐1 receptor agonists bind to GLP‐1 receptors present in the EAT (G. Iacobellis et al. 2017). Microarray analysis of the genes expressed in the EAT of CAD patients demonstrated that there is a correlation between GLP‐1 receptor expression and genes involved in the “browning” of adipose tissue (Dozio et al. 2019). Therefore, GLP‐1 receptor agonists might promote EAT browning and fat burning by binding to their receptors in the EAT. However, further research is needed to know the precise metabolic pathways of these medications.

There is also a paucity of data regarding the effect of GLP‐1 receptor agonists on cardiac ionic channels and currents. Liraglutide has been shown to mitigate AF (Bohne et al. 2023). Patch clamp analysis on liraglutide‐treated cardiomyocytes from metabolic syndrome rat models showed reduced APD and QRS intervals, and it reversed the detrimental changes in Ca‐handling proteins and structural changes in the SR, such as tubule dilations (Durak et al. 2022). However, no significant difference in the risk of various types of arrhythmias in T2DM patients receiving GLP‐1 receptor agonists was observed in a pooled meta‐analysis of clinical trials (Wei et al. 2022).

Nevertheless, the Food and Drug Administration (FDA) has not yet approved any EAT‐lowering medications for obesity‐induced cardiac arrhythmias, thus necessitating more investigations of the underlying cellular mechanisms of these therapeutic approaches and investigations of other possible modulators of metabolic pathways as novel treatment options, such as the LTB4 pathway. Therefore, it is proposed that LTB4‐lowering medications such as zileuton be assessed for EAT‐lowering effects.

## 7 Conclusions and Future Directions

The prevalence of obesity and associated diseases such as T2DM is growing rapidly; therefore, their contribution to cardiac arrhythmias and SCD is becoming more significant. Despite the advances made over the years, therapeutic approaches have not yet fully addressed this matter, as many patients with these conditions continue to suffer from lethal arrhythmias. This, therefore, necessitates further assessment of the underlying metabolic pathways. EAT has emerged as a key inflammatory substrate for cardiac inflammation due to its proximity to the myocardium and its shared circulation. Among lipid‐associated pathways, lipid mediators are an understudied pathway with vital roles in adipogenesis, including both proliferation and hypertrophy of adipocytes. LTB4 is a potent chemoattractant that has recently emerged as a potential factor contributing to cardiac arrhythmias in diabetic patients and preclinical models, but its underlying cellular mechanisms are still not fully understood.

Our review focuses on the biosynthesis of LTB4 and the major mechanisms behind LTB4's detrimental effects in obesity and metabolic conditions, which may contribute to cardiac arrhythmias. Molecules attributed to both upstream and downstream stages of the LTB4 biosynthesis pathways are potentially involved in cardiac Ca storage and decay and overall intracellular Ca regulation, adipogenesis, and IR. In addition to IR, it is hypothesized that LTB4 may be at least partly responsible for the transformation of EAT into an inflammatory substrate by inducing adipocyte hypertrophy, monocyte trafficking, macrophage inflammatory polarization, FFA secretion, and lipid accumulation inside macrophages. Understanding the metabolic pathways underlying LTB4's relationship with EAT paves the way for the development of novel therapeutic approaches to reducing this ectopic fat depot. It is also recommended to (1) conduct future cohort studies including EAT samples from patients undergoing surgery to investigate the effect of the inhibition of the LTB4 biosynthesis pathway at each stage, (2) perform adoptive transfer of immune cells derived from EAT samples of zileuton‐receiving patients into cardiomyocytes of preclinical animal models, and (3) if the appropriate preclinical findings are gathered, organize and conduct clinical trials of obese and diabetic patients to assess the outcomes of short‐term and long‐term zileuton administration on EAT volume. These findings could pave the way for reaching novel therapeutic approaches to alleviating obesity‐induced cardiac arrhythmias.

## Author Contributions

P.B. wrote and finalized the manuscript. K.A.A. undertook manuscript editing and approval and finalized the manuscript. A.S.A. obtained funding and conceived and wrote the paper. All authors have read and agreed to the published version of the manuscript.

## Ethics Statement

The authors have nothing to report.

## Consent

The authors have nothing to report.

## Conflicts of Interest

The authors declare no conflicts of interest.

## Supporting information

Supporting information.

## Data Availability

The authors have nothing to report. All of the relevant data are included within the paper itself.
