# Supplementary material for: Proarrhythmic Lipid Inflammatory Mediators: Mechanisms in Obesity Arrhythmias
Source: J Cell Physiol. 2025 Feb 12;240(2):e70012. doi: 10.1002/jcp.70012 (PMC11822244; doi:10.1002/jcp.70012)
Supplement: Supplementary file 1 — Supporting information. [file JCP-240-0-s001.doc]

Supplemental Table S1. Clinical studies on the role of SGLT inhibitors and GLP-1 receptor agonists on EAT

| **Drug*** | **Pathology** | **Mean BMI** | **Comparative** | **Treatment** | **EAT Analysis** | **EAT (change)** | **Other fat depots** | **Other findings** | **Ref** |
| --- | --- | --- | --- | --- | --- | --- | --- | --- | --- |
| **SGLT2 inhibitors** |  |  |  |  |  |  |  |  |  |
| Empagliflozin  10 mg/day | T2DM  (n=51) | Intervention:  32.6  Control:  34.1 | Placebo | 12 w | MRI | ↔ | ↓VAT | ↔LVMI  ↔LVEF | (Gaborit et al., 2021) |
| Empagliflozin  10 mg/day | HFrEF  (non-DM)  (n=62) | Intervention:  29±6  Control:  30±6 | Placebo | 24 w | MRI | ↓ (5.14± 3.10 mL) | NR | ↓TNF family  ↓E-selectin  ↓Interstitial myocardial fibrosis | (Requena-Ibáñez et al., 2021) |
| Dapagliflozin  5 mg/day | T2DM +CAD  (n=35) | Intervention: 26.2±4.8  Control:  25.1±3.3 | Conventional Tx | 24 w | CT scan | ↓ (15.2±12.8 cm3) | NR | ↓TNF-α  ↓P-wave dispersion  ↓P-wave variation  ∆P-wave dispersion correlated with ∆EAT | (Sato et al., 2020) |
| Dapagliflozin  5 mg/day 4w + 10 mg/day rest of the study | T2DM + BMI ≥ 27 + only treated with metformin  (n=84) | Intervention: 36.6±7.8  Control:  34.7±6 | Placebo | 24 w | Echo | ↓ (1.7 mm, 20%) | NR | - | (Iacobellis & Gra-Menendez, 2020) |
| Dapagliflozin 10 mg/day | T2DM  (n=53) | 34.5 ± 4.7 | Baseline | 24 w | Echo  CT scan | ↓ (17.1 cm3, and 2.94 mm) | ↔L4 vertebral fat | ↔LVEF | (Braha et al., 2019) |
| Dapagliflozin  10 mg/day | T2DM + stable CAD  (n=14) | Intervention:  29.1 ± 1.2  Control:  27.8 ± 1.1 | Placebo | 4 w | PET/CT | ↓ (19%) | ↔Pre-renal fat  ↔Mediastinal fat | ↓Glucose uptake | (Cinti et al., 2023) |
| Dapagliflozin  10 mg/day | T2DM  (n=25) | 26.29 ± 1.95 | Baseline | 24 w | Echo | ↓ (0.93 mm) | NR | ↔LVEF | (Song, Wei, Rui, & Fan, 2023) |
| Dapagliflozin  10 mg/day | T2DM + acute MI (dapagliflozin treatment started after PCI)  (n=49) | Intervention:  27.5  Control:  26.15 | Placebo | 48 w | CT scan | ↔ | NR | - | (Macías-Cervantes, Martínez-Ramírez, Hinojosa-Gutiérrez, Córdova-Silva, & Rios-Muñoz, 2024) |
| Canagliflozin  100 mg/day | T2DM  (n=13) | 27 ± 5 | Baseline | 24 w | Echo | ↓ (2 mm) | ↓VAT | - | (Yagi et al., 2017) |
| Luseogliflozin  2.5 mg/day** | T2DM  (n=19) | 28.7 ± 2.7 | Baseline | 12 w | MRI | ↓ (6 cm3) | ↔VAT | ↓IR | (Bouchi et al., 2017) |
| **GLP-1 Receptor Agonist** |  |  |  |  |  |  |  |  |  |
| Liraglutide  Up to 1.5 mg/day | T2DM + BMI≥27  (n=95) | Intervention:  37.8±67.3  Control:  32.6±6.7 | Metformin monotherapy | 6 m | Echo | ↓ (3.4 mm, 36%) | NR | ↓iLVM | (Iacobellis, Mohseni, Bianco, & Banga, 2017) |
| Liraglutide  Initially, 0.6 mg/day and increased to 1.2 mg/day  if tolerable | Poorly controlled T2DM  (n=21) | 30.97 ± 4.04 | Baseline | 3 m | MRI | ↓ (1.05 mm) | NR | - | (Zhao et al., 2021) |
| Liraglutide  1.8 mg/day | T2DM + BMI≥25  (n= 49) | Intervention:  32.6 ± 4.4  Control:  31.6 ± 3.4 | Placebo | 26 w | MRI | ↔ | ↔VAT | ↔ Adiponectin  ↔Myocardial TG content  No correlation between EAT changes and LV function indices | (Bizino et al., 2020) |
| Liraglutide  1.8 mg/day | South Asian T2DM  (n=47) | Intervention:  30.4 ± 3.8  Control:  28.6 ± 4.0 | Placebo | 26 w | MRI | ↔ | ↓VAT | ↔Myocardial TG content | (van Eyk et al., 2019) |
| Liraglutide (1.2 mg/day) or Exenatide (5 mcg bid 1^st^ month and then 10 mcg bid)  *** | Poorly controlled T2DM  (n=25) | 35.2 ± 4.8 | Baseline | 3 m | Echo | ↓ (NR) | ↓Peri-renal fat  ↓Peri-aortic fat  ↓Peri-umbilical  ↓Sub-xyphoideal | - | (Morano et al., 2015) |
| Exenatide  (5 mcg bid 1^st^ month and then 10 mcg bid) | Poorly controlled T2DM  (n= 44) | Intervention:  37.2 ± 1.8  Control:  35.0 ± 1.2 | Reference treatment | 26 w | MRI | ↓(8.8 ±2.1%) | NR | ↔Myocardial TG content | (Dutour et al., 2016) |
| Semaglutide  Up to 1 mg/w | Obese T2DM | Semaglutide:  34.3 ± 5  Metformin+diet: 33.5 ± 6 | Baseline | 3 m | Echo | ↓: 0.5 mg/w  ↓↓: 1 mg/w | NR | - | (Iacobellis & Villasante Fricke, 2020) |
| Dulaglutide  Up to 1.5 mg/w | Obese T2DM | Dulaglutide:  36.5 ± 6  Metformin+diet: 33.5 ± 6 | Baseline | 3 m | Echo | ↓: 0.75 mg/2  ↓↓: 1.5 mg/w | NR | - | (Iacobellis & Villasante Fricke, 2020) |

↑, Increased; ↓, decreased; ↔, no significant change by the drug; NR, not reported; ∆, change; BMI, body mass index; EAT, epicardial adipose tissue; SGLT2, sodium-glucose co-transporter 2; MRI, magnetic resonance imaging; VAT, visceral adipose tissue; LVMI, left ventricular mass indexed to body surface area; iLVM, left ventricular mass indexed to height ^2.7^; LVEF, left ventricular ejection fraction; HFrEF, heart failure with reduced ejection fraction; DM, diabetic mellitus; TNF, tumor necrosis factor; CAD, coronary artery disease; CT, computer tomography; w, week; m, months; L4, 4^th^ lumbar vertebrae; PET, positron emission tomography; IR, insulin resistance; TG, triglyceride, bid= twice daily, n=number of patients

*Medication is added to the standard therapy that the patients were receiving prior to the study.

**If HbA1c remained over 7%, the dosage was allowed to be increased to 5 mg.

***The results are pooled.

Supplemental Table S2. Electrophysiological changes induced by anti-diabetic medications.

| **Drug** | **Animal model** | **Dose/Concentration and mode** | **Tissue/Cell** | **Pathologic changes** | | **Drug effects** | | **Ref** |
| --- | --- | --- | --- | --- | --- | --- | --- | --- |
| Empagliflozin | STZ-induced T1DM rat (WR) | 10 mg/kg/day po  4 weeks | Ventricle | ↑QTc  ↑Late *I_Na_*  ↓reverse mode NCX  ↑RyR2  ↑Ca decay time  ↑SR Ca content | ↓*I_Ca,L_*  ↑SERCA2a  ↑Ca sparks  ↓Cai-T  ↑APD | ↓QTc  ↓Late *I_Na_*  ↑reverse mode NCX  ↑RyR2  ↓Ca decay time  ↑SR Ca content | ↑*I_Ca,L_*  ↑SERCA2a  ↓Ca sparks  ↑Cai-T  ↓APD | (Lee et al., 2019) |
|  |
|  | STZ-induced T1DM rat (WR) | 1µM in vitro | Ventricle | ↑Late *I_Na_* | ↑APD | ↓ Late *I_Na_* | ↓APD | (Lee et al., 2019) |
|  | Zebra fish (Danio rerio) | 5µM in vitro | Ventricle | NA |  | ↔ Late *I_Na_*  *↑I_K_*_s_  ↓APD | ↔*I_Ca,L_*  ↑*I_K_*_r_ | (Karpushev et al., 2022) |
|  | TAC-induced HF (pressure overload) C57BL/6 mice | 1 or 10 µmol/L  in vitro | Ventricle | ↑Late *I_Na_*  ↑spontaneous  Ca transients* |  | ↔Peak *I_Na_ (*10 µmol/L) | ↓Late *I_Na_ (*10 µmol/L) | (Philippaert et al., 2021) |
|  | Healthy C57Bl/6N mice | 1 µmol/L in vitro | Cardiomyocytes + veratridine (to induce late *I_Na_ )* | ↑spontaneous  Ca transients* |  |  | ↓spontaneous Ca transients* | (Philippaert et al., 2021) |
|  | TAC-induced HF (pressure overload) C57BL6/J mice | 10 μM concentration  in vivo over 4 w | Whole heart | ↑Nav1.5  ↔NCX  ↑APD | ↓ Late *I_Na_*  ↑Ca decay time | ↓Nav1.5  ↔NCX  ↓APD | ↓ Late *I_Na_*  ↓Ca decay time | (Wen et al., 2024) |
|  | HFD-fed db/db mice (severe diabetes) | 150 mg/kg concentration  in vivo over 4.5 w | Ventricle | ↓P-RyR2 | ↔SERCA | ↓↓P-RyR2 | ↔SERCA | (Moellmann et al., 2020) |
|  | Aldosterone induced HFpEF db/db mice | 1 μmol/L in vitro | Ventricle | ↑Late *I_Na_* (male>female)  ↓*I_K_*_1_ (male+ female)  ↑APD  (male+ female) | ↓*I_Ca,L_* (male)  ↓*I_K_*_s_ (male)  ↓*I_to_* (male+ female)  ↑DAD  (male+ female) | ↓APD (male+ female) | ↓DAD (male+ female) | (Mira Hernandez et al., 2024) |
|  | HFpEF C57BL/6J mice | Acute 1 μmol/L (3 min) | Ventricle | ↑Late *I_Na_* |  | ↔Late *I_Na_* |  |  |
|  | HFpEF C57BL/6J mice | Pre-incubation for 4 h | Ventricle | ↑Late *I_Na_* | ↑APD | ↓Late *I_Na_* | ↓APD | (Hegyi et al., 2022) |
| Dapagliflozin | Xenopus laevis frogs | 100 µM in vitro | Oocytes with heterologous expression of K channels** | NA |  | *↓I*_Kur_  *↑I*_K1_  *↔I_to_* | ↓*I_K_*_r_  *↑I*_K2p_ 17.1 current  ↔K_2P_3.1 | (Hegyi et al., 2022) |
| Liraglutide | db/db  C57BL/6J mice | 0.25 mg/kg/d sc 4w | - (only in vivo ECG analysis for liraglutide study) | ↓HR  ↑AERP  ↑cSNRT  ↔QRS | ↑AF susceptibility  ↑P-wave duration  ↑P-R interval  ↑AVERP  ↔QT interval | ↔HR  ↔AERP  ↔cSNRT  ↔QRS | ↔AF susceptibility  ↓ P-w duration  ↔P-R interval  ↓AVERP  ↔QT interval | (Müller et al., 2024) |
|  | Sucrose induced  MetS rats (WR) | 0.3 mg/kg 4w | Ventricle | ↓HR  ↑APD  ↔NCX protein  *↓I_to_*  T-tubule dilation | ↑P-R interval  ↑Peak *I_Na_*  ↑INCX  ↑RyR2 Ca leak  ↑pRyR | ↑HR  ↓APD  ↔NCX protein  *↑I_to_*  ↓T-tubule dilation | ↓P-R interval  ↔Peak *I_Na_*  ↑INCX  ↓RyR2 Ca leak  ↔pRyR | (Bohne et al., 2023) |
|  |  |  |  |  |  |  |  | (Durak et al., 2022) |

↑, Increased; ↓, decreased; ↔, no change; STZ, streptozotocin; T1D, type 1 diabetes; po, by mouth; WR, Wistar Rats; QTc, corrected QT interval; Late *I_Na_*, late sodium current; NCX, sodium-calcium exchanger; RyR2, ryanodine receptor 2; SR, sarcoplasmic reticulum; *I_Ca,L_*, L-type Ca current; SERCA, sarcoplasmic/endoplasmic reticulum Ca2+-ATPase; Ca_i_-T, intracellular Ca transient; APD, action potential duration; *I_Ks_*, Slow component of the delayed rectifier potassium current; *I_Kr_*, Rapid component of the delayed rectifier potassium current; *db*/*db*, a genetically diabetic mouse; P-RyR, phosphorylated RyR, HFD, IK1, Inwardly rectifying potassium current; *I_Kur_*, Ultra-rapid outward potassium current; Ito, transient outward potassium current; HFpEF, heart failure with preserved ejection fraction; HR, heart rate; AF, atrial fibrillation; AERP, atrial effective refractory period; AVERP, atrioventricular Effective Refractory Period; cSNRT, corrected Sinus Node Recovery Time; T-tubule, transverse tubule

*Induced by veratridine.

**K channels expressed included KCNH2, KCNA5, KCND3, KCNJ2, KCNK2, KCNK3, and KCNK1.

**REFERENCES**
